# The Toxicity and Metabolism Properties of* Herba Epimedii* Flavonoids on Laval and Adult Zebrafish

**DOI:** 10.1155/2019/3745051

**Published:** 2019-03-03

**Authors:** Rongling Zhong, Ying Chen, Jie Ling, Zhi Xia, Yang Zhan, E. Sun, Ziqi Shi, Liang Feng, Xiaobin Jia, Jie Song, Yingjie Wei

**Affiliations:** ^1^Affiliated Hospital of Integrated Traditional Chinese and Western Medicine, Nanjing University of Chinese Medicine, Nanjing, Jiangsu, China; ^2^Key Laboratory of Delivery Systems of Chinese Materia Medica, Jiangsu Provincial Academy of Chinese Medicine, Nanjing, Jiangsu, China

## Abstract

Zebrafish is being increasingly used for metabolism and toxicity assessment. The drugs consumed in zebrafish metabolism studies are far less than those used in rat studies. In our study, zebrafish embryos were exposed to icariin, Baohuoside I (BI), Epimedin A (EA), Epimedin B (EB), Epimedin C (EC), Sagittatoside A (SA), Sagittatoside B (SB), and 2′′-O-rhamnosylicariside II (SC), respectively, to examine the toxicity and metabolic profiles of these flavonoids. The order of toxicity was SC, SB > EC, SA > BI, icariin, EA, EB. After 24 h exposure to SB and SC, the mortality of zebrafish larvae reached 100% and yolk sac swollen was obvious. Both SC and SB caused severe hepatocellular vacuolization and liver cells degeneration in adult zebrafish after 15 consecutive days' treatment. The metabolic profiles of these flavonoids with trace amount were also monitored in larvae. BI was the common metabolite shared by icariin, EA, EB, SA, and SB, via deglycosylation. Both BI and SC remained as the prototype in the medium, suggesting that it is hard for BI and SC to cleave the rhamnose residue. EC was metabolized into SC and BI in zebrafish, inferring that SC might be responsible for the toxicity observed in EC group. The metabolites of icariin, EA, EB, EC, and BI in zebrafish larvae coincided with results from rats and intestinal flora. These data support the use of this system as a surrogate in predicting metabolites and hepatotoxicity risk, especially for TCM compound with trace amount.

## 1. Introduction

*Herba Epimedii* known as YinYangHuo in Chinese is the dried leaf of the* Epimedium* and has been historically used in combination with other herbs to treat skeletal diseases in traditional Chinese medicine (TCM) [1]. Metabolic pathways of other* Epimedium* flavonoids, namely, Epimedin A (EA) [2], Epimedin B (EB) [3], Epimedin C (EC) [4], icariin [5], and Baohuoside I (BI) [6], were well described in both mammals and adult zebrafish (*Danio rerio*) [7]. However, no data have been reported on the metabolism of* Epimedium* flavonoids such as Sagittatoside A (SA), Sagittatoside B (SB), and 2′′-O-rhamnosylicariside II (SC), probably attributed to the difficult separation and purification process. Metabolic pathways observed in adult zebrafish are consistent with those obtained from rats, mainly involving deglycosylation, hydrogenation, hydroxylation, and glucuronidation. Drugs consumed in adult zebrafish metabolism studies equal approximately 2 mg, far less than those used in rat studies [7]. Therefore, zebrafish model is preferred for metabolic research of such trace compounds isolated from TCM.

The genomes of zebrafish share approximately 85% homology with humans [8] and their molecular and morphological basis of tissue and organ development is very similar to humans [9]. Easy maintenance, high proliferation (each adult female fish can lay about 300 eggs every week) [10], transparence up to 14 days, low cost, and convenient drug delivery make zebrafish an ideal model for exploring biological processes [11, 12]. Zebrafish model can predict the metabolism and excretion of chemicals from TCM, such as ibuprofen [13] and asperosaponin VI [14]. A large number of phase I and phase II metabolizing enzymes, along with intestinal flora in zebrafish larva, are sharing a strong analogy to humans', such as CYP2B6, 1A1, 3A4, 2E1, sulfotransferase, glucuronosyl-transferase, and glutathione-S-transferase [15]. When exposed to drug solutions, zebrafish can absorb compounds continuously from water via the cavity or the skin. The metabolites transformed by zebrafish will finally be excreted into the water.

Zebrafish developed functioning organs and tissues within 5 days after fertilization (dpf), including liver, heart, brain, bone, intestines, kidney, sensory organs, and nerve systems [16]. Equipped with the obvious advantages in identifying endpoints of toxicity [17], zebrafish larva is regarded as a highly predictive model for rapid evaluation of hepatotoxicity* in vivo*. Specifically, hepatocytes are already present from 2 dpf onwards. A transcriptomics-based hepatotoxicity comparison between the zebrafish embryo and traditional models (primary human hepatocytes,* in vitro *rat hepatocytes and* in vivo* rat liver) exhibited concordance in the metabolic pathway [18].

*Epimedium* is considered to be relatively safe and no major side effects have been discovered in acute or chronic toxicity studies [19]. Recently, a Chinese herbal prescription has been reported to cause acute liver failure [20]. Two elderly women suffering from osteoporosis developed significant elevation of alanine amino transferase (ALT) and aspartate amino transferase (AST) after taking XLGB capsules for several months [21]. And* Epimedii *occupies 70% of this prescription by content [22]. As reported by Chen et al. [23], vomit, nausea, and motion decrement were observed in mice after 3-day administration of* Epimedium*. Even worse, liver steatosis occurred after 15-day administration of* Epimedium*. The author speculated that the androgen-like effect of* Epimedium* is closely related to the hepatotoxicity. Moreover, Baohuoside I was a major metabolite of* Epimedium* extract. The cytotoxic effect of Baohuoside I has been proved in numerous research. For instance, Baohuoside I decreased the viability of human hepatoblastoma HepG2 cells in a concentration- and time-dependent manner [24]. Although there are no known warnings or contraindications for the use of* Epimedium* plants, a more rigorous safety evaluation is needed to determine any potentially harmful effects.

Here, we assessed the metabolism and toxicity of* Epimedium* flavonoids by zebrafish model, aiming at (a) screening hepatotoxicant and illustrating the metabolic pathways of these flavonoids and unearthing the close relationship between metabolism and toxicity and (b) comparing the results with literatures and reinforcing the application of zebrafish in these fields.

## 2. Materials and Methods

### 2.1. Chemicals and Reagents

Icariin (purity > 98%) was obtained from the National Institute for Food and Drug Control (Beijing, China), while Baohuoside I (BI), Epimedin A (EA), Epimedin B (EB), Epimedin C (EC), Sagittatoside A (SA), Sagittatoside B (SB), and 2′′-O-rhamnosylicariside II (SC) were isolated and purified in our laboratory [2]. The purity (all > 98%) was determined by HPLC. Dimethyl sulphoxide (DMSO) were purchased from Sigma-Aldrich (St. Louis, MO, USA). Test drugs were dissolved in DMSO or ddH_2_O and serial dilutions were made before experiments. HPLC-grade acetonitrile and methanol were purchased from Tedia Company (Fairfield, CT, USA). 0.5% DMSO or fish water was used as vehicle control. The concentration of DMSO keeps equal to or less than 0.5% because 0.5% DMSO had no effect on morphology and viability of zebrafish embryos (data not shown). Ultrapure water was produced using a Millipore Milli-Q Gradient system (Milford, MA, USA). All reagents were of analytical grade or better.

### 2.2. Zebrafish Husbandry

A wild-type Tubingen strain of zebrafish was supplied by Model Animal Research Center of Nanjing University (Nanjing, China). Adult zebrafish were maintained at 28°C, in a 12 h light and 12 h dark cycle conditions. The treatment was approved by the Animal Ethics Review Committee, Jiangsu Provincial Academy of Chinese Medicine. Embryos were collected immediately after natural spawning and cultured at 28°C for 24 h in egg water (5 mM NaCl, 0.17 mM KCl, 0.4 mM CaCl_2_, and 0.16 mM MgSO_4_) (Komoike and Matsuoka, 2013). Because the yolk sac can supply nutrition to zebrafish embryos, no feeding is requisite during the initial 9 to 10 dpf. This study and the experimental protocols were approved by the Institutional Animal Care and Use Committee (IACUC) of Jiangsu Provincial Academy of Chinese Medicine.

### 2.3. The Toxicity Assays on Larval Zebrafish

In order to evaluate the potential toxicity of* Epimedium* flavonoids, zebrafish larvae (1 dpf, 24 h after fertilization) were exposed to DMSO vehicle, icariin, BI, EA, EB, EC, SA, SB, and SC (5 to 200 *μ*M) in the egg culture water from 1 dpf to 6 dpf. Drug exposure was performed in 24-well plates, with 8 larvae in each well and two well replicates. The treatments were performed thrice independently. Major zebrafish organs and tissues were visually assessed. The fish were placed on glass slide for visual observation and image acquisition under a microscope. The morphology was under observation, and mortality of larval zebrafish was recorded every day. The observer was blinded from the separate exposure groups.

### 2.4. Metabolism of* Epimedium* Flavonoids on Zebrafish Larvae

To evaluate the metabolism of* Epimedium* flavonoids, zebrafish larvae were exposed to DMSO vehicle, icariin, BI, EA, EB, EC, SA, SB, and SC (25 *μ*M) from 1 dpf to 6 dpf. Drug exposure was performed in 24-well plates, with 8 larvae in each well and three well replicates. The medium was collected to determine the metabolites at 1 dpf, 2 dpf, 3 dpf, 4 dpf, 5 dpf, and 6 dpf, respectively. The sample solution was evaporated to dryness with vacuum at room temperature. 80% methanol were added to dissolve the residue and further centrifuged at 12,000 rpm for 15 min. The supernatant (20 *μ*L) was subjected to high performance liquid chromatography (HPLC) analysis with a Zorbax C-18 column (5 *μ*m, 150 mm × 4.6 mm). The mobile phase consisting of 0.05% formic acid in water (A) and 0.05% formic acid in acetonitrile (B) was pumped at a flow rate of 1.0 mL/min. The column temperature was set at 30°C. The gradient elution program was set as follows: 0–5 min, 10% B; 5–15 min, 10–25% B; 15–40 min, 25–90% B; 40–45 min, 90% B.

### 2.5. Histology Examination for Liver Toxicity

To further examine the potential liver toxicity of flavone, adult zebrafish of mixed genders were used for histology examination on liver slices. The fish were treated with icariin, BI, EC (50 *μ*M, respectively) or SC and SB (25 *μ*M, respectively) for 15 days, with five fish in each group. Drug exposure was performed in small size brown fish tanks, with 50 mL incubation medium for per adult fish. To avoid the growth of microorganisms, solutions containing test drug were replaced every two days. At the end, the fish were sacrificed by embedding them in ice and subsequently fixed in 4% paraformaldehyde according to standard procedures. Sections (5 *μ*m) were stained with hematoxylin and eosin (H&E) as previously reported [25] and analyzed for general structural changes in the liver with light microscopy.

### 2.6. Statistical Analysis

Data were analyzed using Graphpad Prism 5.01 (GraphPad Software Inc., San Diego, CA, USA). Statistical analysis was performed using one-way analysis of variance (ANOVA), followed by posttests. Quantitative data are presented as the mean ± SD. Differences were considered significant at* P* < 0.05.

## 3. Results

### 3.1. Toxicity of* Epimedium* Flavonoids on Zebrafish Larvae

**Figure 1:**
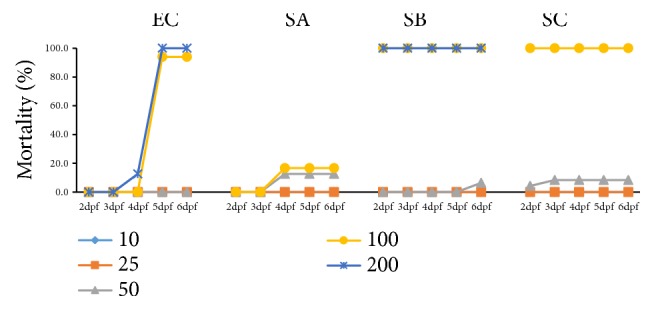
Mortality curves of zebrafish larvae. From 2 dpf to 6 dpf, after exposure to EC, SA, SB, and SC (10, 25, 50, 100, and 200 *μ*M).

Embryos (1 dpf) were exposed to graded concentrations (5-200 *μ*M) of BI, icariin, EA, EB, EC, SA, SB, and SC for 1-5 days (2-6 dpf). Morphologies of the zebrafish embryos in each group were visually examined under a microscope, such as pericardial edema, crooked body, tail malformation, yolk sac swollen, and spinal deformity. No death or morphological abnormalities were observed in icariin, BI, EA, or EB-treated fish after 1-5 days of exposure, at concentrations up to 200 *μ*M. Likewise, EC at low concentration (50 *μ*M) did not reveal any negative effect on larvae mortality from 2 to 6 dpf. By contrast, the survival rates of larvae (5 dpf) in 100 and 200 *μ*M EC groups dropped to 6% and 0%, respectively. For SA at 100 *μ*M, the mortality rate at 4 dpf was 16.7%. However, the mortality at 2 dpf increased abruptly from 0% to 100% between the concentrations of 50 to 100 *μ*M (SB and SC) after 24 h exposure (Figure 1). Moreover, SB and SC incubation resulted in the whitening and clotting of the larvae. The body length of embryo at 3 dpf was shorter in both SB and SC (50 *μ*M) groups than the control group, suggesting that SB and SC might have developmental toxicity.

**Figure 2:**
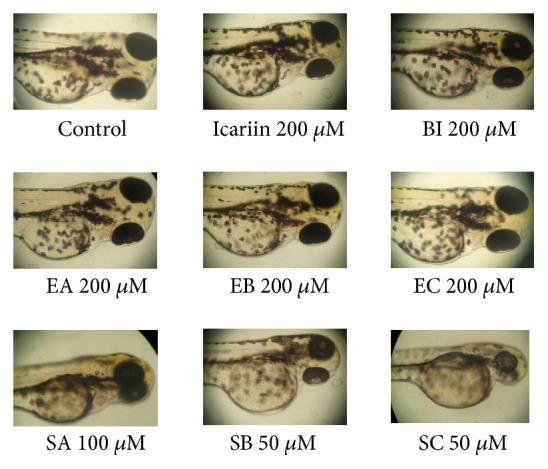
Visual phenotype of yolk sac in zebrafish at 3 dpf after exposure to drugs from 1 dpf to 3 dpf. SB and SC notably delayed the absorption of yolk sac. Normal phenotype of yolk sac was found in icariin, BI, EA, and EB groups, whereas slight yolk sac swollen was found in EC and SA groups. Scale bars = 1,000 *μ*m.

In the phenotype evaluation (3 dpf), both SB and SC (50 *μ*M) notably delayed the absorption of yolk sac (Figure 2), indicating potential hepatotoxicity of these two compounds. These results suggested that icariin, BI, EA, and EB have negligible toxicity for zebrafish embryos, while EC and SA have slight toxicity for the larvae. SB and SC are much more toxic to zebrafish embryos than icariin, BI, EA, EB, EC, and SA, as demonstrated by 100% mortality rate and yolk sac swollen. The order of toxicity was SC, SB > EC, SA > BI, icariin, EA, EB. The maximum nonlethal concentration (MNLC) of these flavonoids in larvae was close to 50 *μ*M, so concentration of 0-50 *μ*M was used for subsequent metabolism and target organ toxicity experiments.

### 3.2. Metabolism of EA, EB, Icariin, SA, SB, and BI on Zebrafish Larvae

**Figure 3:**
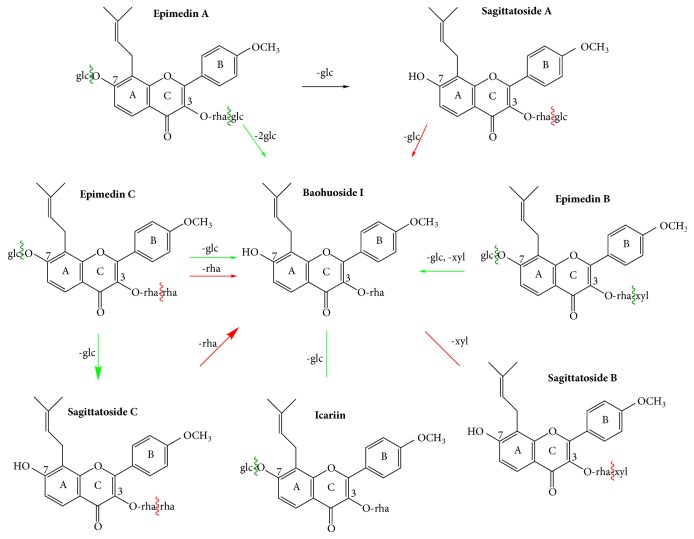
Major metabolic pathway of flavonoids from* Herba Epimedii* on larval zebrafish. BI was the common metabolite shared by icariin, EA, EB, SA, and SB, via one or two steps of deglycosylation. EC was transformed into SC. But it is hard for BI and SC to cleave the rhamnose residue. Green arrow and wavy line mean easy deglycosylation, and red arrow and wavy line mean relatively hard deglycosylation process.

**Figure 4:**
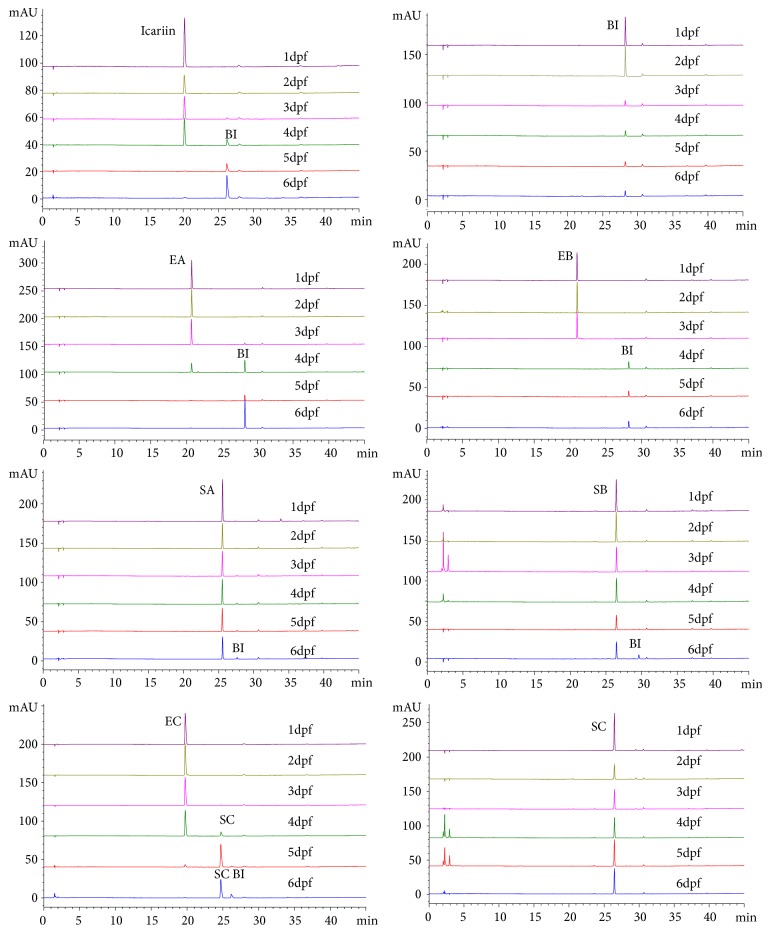
HPLC chromatogram of the metabolism on larval zebrafish. The incubation medium from icariin, BI, EA, EB, EC, SA, SB, and SC groups was analyzed by HPLC at 1 dpf to 6 dpf.

**Figure 5:**
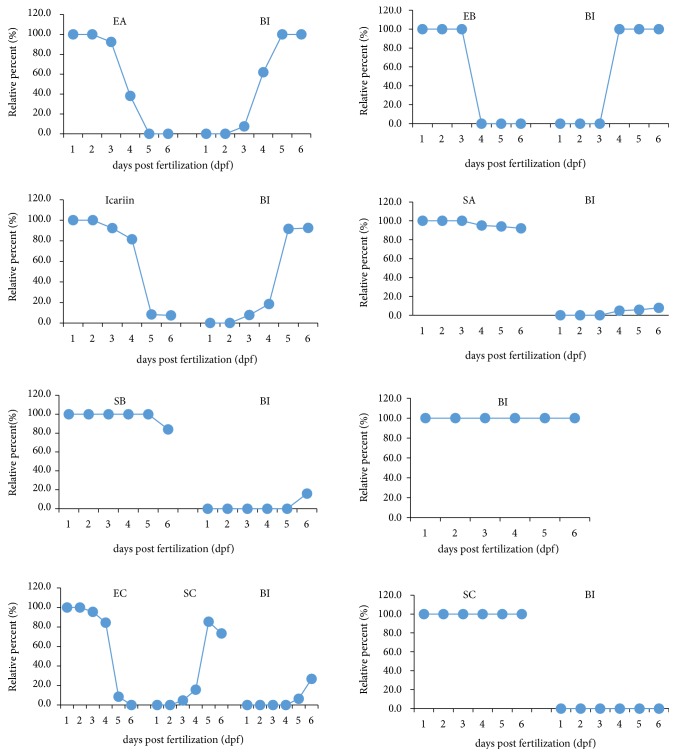
The variation of the contents of the metabolites and the parent compound on zebrafish larvae. In EA, EB, SA, SB, and icariin groups, the content of the metabolite BI was rising. Both BI and SC remained as the prototype in the medium from 1dpf to 6 dpf. In EC group, two metabolites SC and BI were detected.

After 24 h to 120 h exposure, metabolites of flavonoids from* Herba Epimedii* were detected by HPLC analysis with a Zorbax C-18 column. Icariin was transformed into BI via one step of hydrolysis of glucosides. EA was directly transformed into BI via cleavage of both 7-OH and 3-OH glucose residues. Likewise, EB was transformed into BI rather than SB, through cleavage of 7-OH glucose residue and 3-OH xylose residue (Figure 3). Both EA and EB underwent two steps of hydrolysis, and only the final metabolite BI can be observed in the chromatogram (Figures 4 and 5). These results were in accordance with previous reports from* in vivo *rat model and* in vitro* intestinal bacteria metabolism model [26, 27]. At 4 dpf, 67% EA, 100% EB, and 10% icariin were metabolized into BI. At 5 dpf, both EA and icariin were entirely transformed into BI. In the medium of BI-treated group, only the prototype of BI was detected, even after 5 days of treatment (6 dpf). No other metabolites were yielded, suggesting that rhamnose residue at the 3-OH of ring C is hardly eliminated from BI.

Glucose or xylose residue on the 3-OH of ring C was cleaved from SA and SB, generating the same metabolite BI. But the deglycosylation rate of SA and SB at 3-OH is much slower than the hydrolysis speed for EA, EB, and icariin. In detail, only a small amount of BI was found in the medium of SA group until 72 h incubation (4 dpf), and the content of BI merely reached 7.9% at 6 dpf. For SB group, BI was not detected until 6 dpf. In summary, BI was the common metabolite shared by icariin, EA, EB, SA, and SB, via fast or slow hydrolysis steps.

### 3.3. Metabolism of EC and SC on Zebrafish Larvae

Within the initial 48 h treatment (from 1 dpf to 3 dpf), EC were mainly excreted as the prototype (Figures 4 and 5). The metabolite SC could not be found in the medium of the EC zebrafish larvae group until 72 h incubation (4 dpf). By comparing the retention time of the reference standard compound, the peak was identified as SC, forming via the cleavage of glucose residue at the 7-OH. At 4 dpf, 20% of EC was transformed into SC and the transformation rate reached 92% at 5 dpf. A small amount of BI can be detected in the solution of EC group at 5 dpf, probably via the elimination of rhamnose residue at the 3-OH from SC. The relative content for SC and BI was 85.3% and 6.2% at 5 dpf, respectively. At 6 dpf, EC prototype could not be detected anymore in the solution, and the relative content for SC and BI changed into 73.3% and 26.7%, respectively. It can be inferred that a portion of the metabolite SC has been further metabolized into BI.

When it comes to the metabolic pathway of SC, unexpectedly, only SC prototype was detected in the medium from 1 dpf to 6 dpf, without generating the metabolite BI. But the transformation of SC into BI at 5 dpf and 6 dpf was observed in EC-treated group. We speculated that incubation with EC from 1 dpf to 5 dpf has an impact on the development of the intestine and CYP450 enzyme system in larvae, thus promoting the transformation of SC into BI. This might explain the different phenomenon in EC and SC groups.

### 3.4. Hepatotoxicity of SB and SC on Zebrafish Adults

Liver histopathology evaluations on adult zebrafish were conducted to evaluate the hepatotoxicity of SA, SB, SC, EC, BI, and icariin. Given that zebrafish embryos are well tolerated to EA and EB treatment and both of them are mainly metabolized into BI, evaluations on BI hepatotoxicity can also elucidate whether EA and EB are safe to the larvae. The MNLC dose (50 *μ*M) was chosen for histology study on zebrafish adults. However, in both SB and SC groups at 50 *μ*M, zebrafish adults died within 30 min incubation. Herein, a lower concentration of 25 *μ*M for SB and SC was chosen, in order to examine the liver toxicity after long-term treatment. All the fish survived till the end of the 15-day treatment.

**Figure 6:**
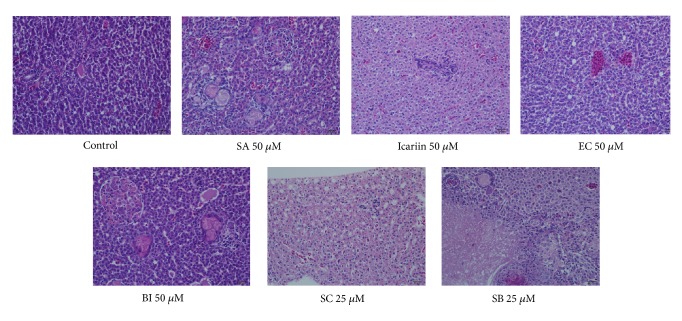
H&E staining of liver sections from adult zebrafish. The representative figures indicate normal liver in vehicle group as well as icariin, EC, and BI (50 *μ*M) groups after 15-day treatment. Liver degeneration, hepatocellular vacuolization, and infiltration of inflammatory were found in zebrafish treated with SB and SC (25 *μ*M).

As demonstrated in H&E staining (Figure 6), zebrafish liver in the untreated and vehicle (0.5% DMSO) group showed the structural integrity of the hepatic cell. Liver section of zebrafish exposed to SA, BI, and icariin (50 *μ*M) for 15 days appeared similar to the vehicle group. No apparent changes were observed in hepatocytes in EC group (50 *μ*M), except slight edema and negligible vacuolization. In SC-treated group (25 *μ*M), the liver sections showed severe hepatocellular vacuolization, mild degeneration of liver cells, edema, and slight infiltration of inflammatory and, while SB (25 *μ*M) caused a certain degree of cells necrosis, moderate edema with mild vacuolar changes, loose cell-to-cell contact, lymphocyte and mononuclear infiltration, and Kupffer cell proliferation in mesenchyme, suggesting that SB and SC are hepatotoxic even at lower concentrations.

## 4. Discussions

The zebrafish, as a model organism, is being increasingly used for pharmaceutical metabolism. In the present study, zebrafish larva was applied to explore the metabolism of EA, EB, EC, icariin, SA, SB, SC, and BI, after 24 h to 120 h incubation. Before we carried out the metabolism and toxicity study, stability test of these compounds in the medium has been performed. As shown in supplementary Figure (S1), these compounds keep stable till the fifth day. BI was the common metabolite shared by icariin, EA, EB, SA, and SB, via one or two steps of deglycosylation. EC was transformed into SC. The metabolism of SA, SB, and SC was reported for the first time. SA and SB were deprived with glucose or xylose, mainly converting into BI. But it is hard for BI and SC to cleave the rhamnose residue. Both BI and SC remained as the prototype in the medium from 1 dpf to 6 dpf. In general, the metabolic pathways of icariin, EA, EB, EC, and BI in zebrafish larvae coincided with results obtained from rodent model,* in vitro* intestinal flora, and zebrafish adult model [3–7, 27]. In addition, the metabolic pathway of SA, SB, and SC is similar to that of the homologous flavonoids EA and BI, consolidating the application of zebrafish embryo in drug metabolism predicting.

Based on the characteristic of the metabolic pathway,* Epimedii* flavonoids can be divided into two categories. One category includes EA, EB, icariin, SA, SA, and BI, all of which share the same metabolite BI. The deglycosylation speeds for glycoside, xyloside, or rhamnoside from the parent compounds were different among these flavonoids. Glucoside at 3-OH or 7-OH and xyloside at 3-OH were easily and quickly cleaved from icariin, EA, and EB, respectively. But the hydrolysis speed of glucose at 3-OH from SA or xylose at 3-OH from SB slowed down considerably. In contrast, rhamnose residue at 3-OH was hardly eliminated from BI or SA. The other category includes EC and SC, both of which share the same metabolite SC. EC can easily cleave the glucose, transforming into SC. But the elimination of rhamnoside from the metabolite SC in EC-treated group indeed took some time. The transformation of SC into BI was not detected until 5 dpf in EC-treated group.

The applicability of the zebrafish larva in metabolism study further supports the use of this system in toxicity screening. The zebrafish has been widely used for estimating the hepatoxicity of chemicals [28]. The liver is known as an important target organ for xenobiotic chemicals. High concentrations of xenobiotics may end up in the liver after intestinal absorption. Primary hepatocytes, liver slices, and human hepatocyte HepaRG line have been established to measure liver injury [29]. However, the reductionistic nature and the loss of functionality severely limited the wide use of these* in vitro* methods. In next-generation RNA sequencing, strong similarities were found between whole zebrafish embryo and mouse liver in pathways associated with hepatotoxicity. At 3 dpf, the liver of zebrafish is fully functioning, including the CYP450 enzymes, which are essential for metabolizing xenobiotics [30]. In addition, embryogenesis of zebrafish is finished within 5 dpf. Thus, hepatic responses can be expected after exposure to hepatotoxicants in zebrafish embryo.

In this paper, zebrafish larva was used for hepatotoxicity testing of* Epimedium *flavonoids. Among them, SB and SC exerted remarkable hepatotoxicity. When zebrafish larvae were treated with EC (200 *μ*M) for 3 days (4 dpf), no significant toxicity was observed, as revealed by whole fish survival and normal yolk sac morphology. But when the incubation time reached 4 days (5 dpf), the mortality of EC group (both 100 and 200 *μ*M) surged to almost 100%. According to the metabolic results, the generation of SC in the solution of EC group was negligible at 4 dpf. But afterwards, more and more EC was transformed into SC. 92% of EC has been transformed into SC at 5 dpf and EC could not be detected anymore in the medium at 6 dpf. Therefore, we speculated that the metabolite SC might be responsible for the lethal effect of EC after long-time transformation.

When it comes to the hepatotoxicity assessment in adult zebrafish, degeneration of liver cells was found after 15 days of SC treatment (25 *μ*M). However, no obvious liver injury was found after 15 days of EC treatment. According to our previous research, EC was mainly metabolized into SC in adult zebrafish after 24 h exposure, but only a small quality of SC was generated [7], far less than 25 *μ*M. More importantly, in order to avoid the growth of germs in adult fish experiments, the incubation medium containing the test drug was replaced every other day, whereas the incubation solution was not changed throughout the zebrafish larvae experiment. Therefore, it is reasonable that no apparent hepatotoxicity was spotted in EC-treated adult zebrafish group (50 *μ*M) after 15 days of treatment, but 100% mortality of larvae was found in EC group at 5 dpf. In summary, the hepatotoxicity of SB and SC are most distinguished, and the toxicity induced by EC in larva is probably attributed to the metabolite SC.

The metabolic pathways of icariin, EA, EB, EC, and BI in zebrafish larvae coincided with results from rats and intestinal flora. Not only metabolism and excretion, but the consequent changes in toxicity can also be simultaneously elucidated in zebrafish model. This convenient, time-saving, and predictive animal model can serve as an intermediate step between cell-based evaluation and mammalian animal testing.

## Data Availability

The data that support the findings of this study are available from the corresponding author upon reasonable request.

## Supplementary Materials

Supplementary Materials
* Figure S1*. A: HPLC of total flavonoids of* Epimedium* koreanum in the days 0 and 5, respectively. B: HPLC of mixed reference substance, including 1 EA, 2 EB, 3 EC, 4 icariin, 5 SA, 6 SB, 7 SC, and 8 BI.
