# Supplementary material for: The Toxicity and Metabolism Properties of Herba Epimedii Flavonoids on Laval and Adult Zebrafish
Source: Evid Based Complement Alternat Med. 2019 Mar 3;2019:3745051. doi: 10.1155/2019/3745051 (PMC6421038; doi:10.1155/2019/3745051)
Supplement: Supplementary Materials — Figure S1. A: HPLC of total flavonoids of Epimedium koreanum in the days 0 and 5, respectively. B: HPLC of mixed reference substance, including 1 EA, 2 EB, 3 EC, 4 icariin, 5 SA, 6 SB, 7 SC, and 8 BI. [file 3745051.f1.pptx]

## Slide 1
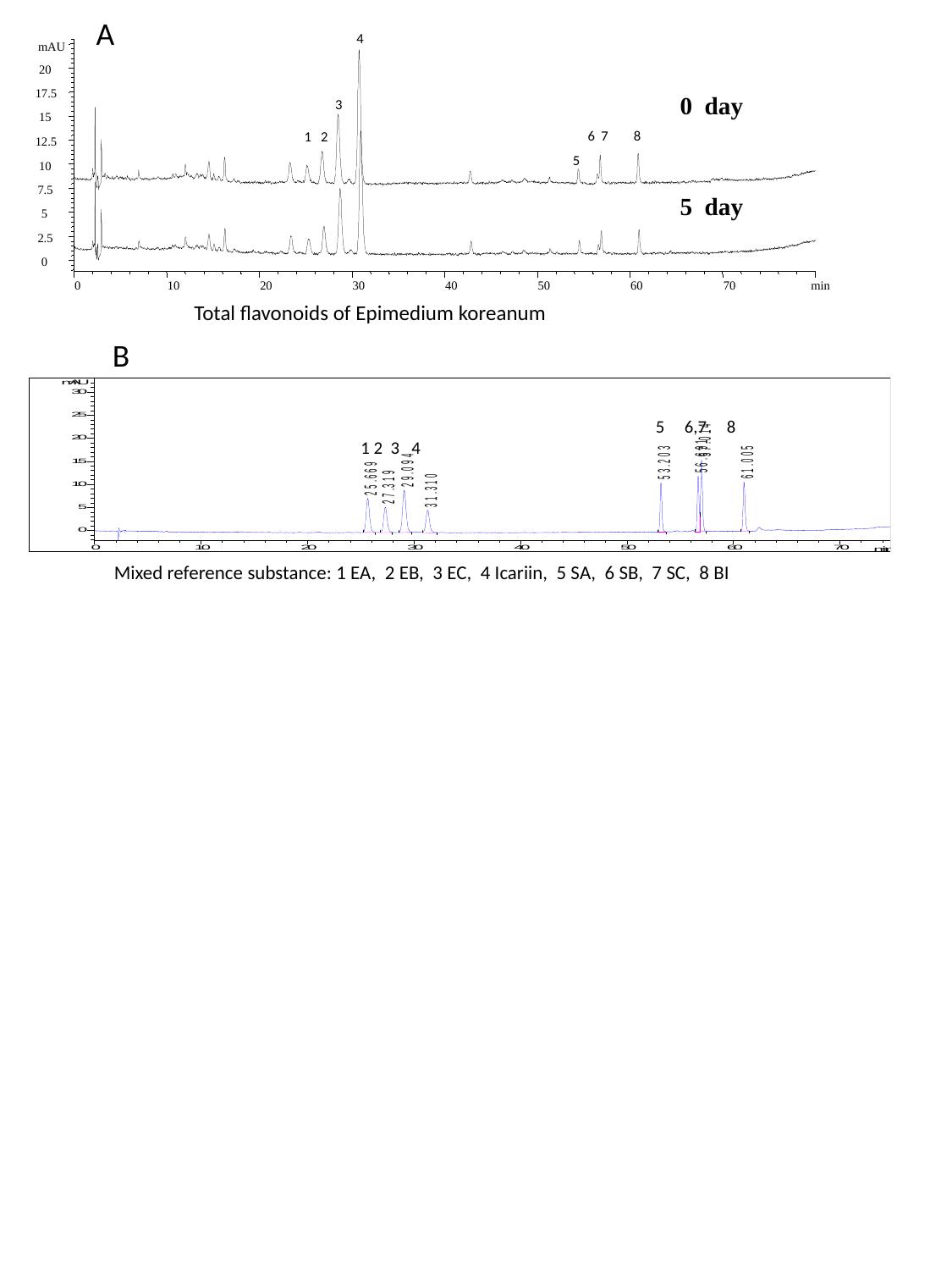

A
4
mAU
20
17.5
15
12.5
10
7.5
5
2.5
0
0
10
20
30
40
50
60
70
min
0 day
5 day
3
 6 7 8
1 2
5
Total flavonoids of Epimedium koreanum
B
 5 6,7 8
1 2 3 4
Mixed reference substance: 1 EA, 2 EB, 3 EC, 4 Icariin, 5 SA, 6 SB, 7 SC, 8 BI
